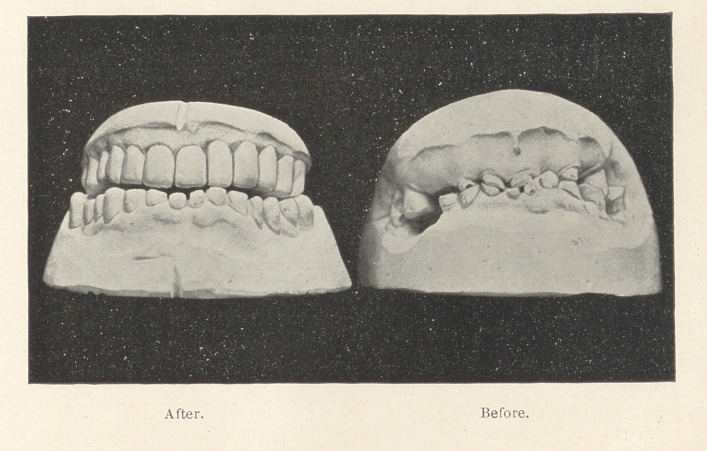# Defective Articulation Accompanied by Pain Corrected by Opening the Bite and the Insertion of Immovable Bridges

**Published:** 1897-07

**Authors:** J. G. Brigiotti

**Affiliations:** Paris, France


### DEFECTIVE ARTICULATION ACCOMPANIED BY PAIN CORRECTED BY OPENING THE BITE AND THE INSERTION OF IMMOVABLE BRIDGES.[1]

BY J. G. BRIGIOTTI,[2] PARIS, FRANCE.

I WAS called upon a year ago to attend a distinguished clergyman, who had the singular habit of moving his jaw from side to side, and forward and backward, until he had worn down his few remaining teeth nearly to the level of the gum.

There were only left in the upper maxillary three incisors, one canine on the left side, and two sixth-year molars.

In the lower jaw there were four incisors, two canines, one left bicuspid, and two twelfth-year molars.

All these teeth, or rather their ruins, were in the most deplorable condition, and the patient suffered much pain, as already intimated.

The conditions can be seen in the model which I have the honor to present to the Institute.

---

[1] Read at the meeting of The New York Institute of Stomatology, March, 1897.

[2] Professor at the École Odontotechnique de Paris.

As can be readily understood, my patient had great difficulty in masticating his food, and his health had suffered greatly in consequence of this defective mastication.

Such was his condition when he first called upon me.

After having examined this interesting case, I determined to make use of the dental remains which I have described above, by adapting to them a strong and immovable apparatus which should enable my patient not only to resume his profession of preaching, but to masticate satisfactorily.

Fearing that a removable apparatus might rather increase than diminish the habit which he had contracted of grinding his teeth, I applied a fixed apparatus, which proved very useful to him and with which we are both perfectly satisfied.

As the two upper molars had no antagonists, there was consequently a tendency to use the forward and backward motion of the lower jaw, which eventually resulted in the projection of the jaw already mentioned.

To correct this defect I thought it would be practicable and advisable to open the bite. To this end I fitted caps to two teeth on each side of the lower jaw and fitted artificial teeth into the spaces between the caps, soldering all together.

These fixtures were then firmly cemented to the teeth. The spaces below were in this way completely filled. After inserting this appliance I waited a fortnight to judge of results and to assure myself as to whether the patient was going to be able to tolerate the new conditions. He seemed quite contented and experienced no inconvenience from the apparatus.

I then proceeded to the second operation. Instead of making a single fixture, I made two separate ones, so as to facilitate repairs should any ever be necessary.

I placed half-caps around the worn incisors, as well as around the cuspid, and two entire caps upon the molars at either side, held firmly together by a gold plate to which they were soldered.

All intervening spaces were then filled by soldering artificial teeth or crowns to these gold plates. These in their turn were cemented to place.

I saw the gentleman three months ago; he was delighted with the result of our work, and had been masticating with so much energy that I was obliged to insert gold fillings into the worn caps.

By comparing the two photographs which I have the honor to present beneath, one can realize the change that has been made. The upper jaw, instead of seeming to be sunken, has resumed its

After.

Before.

After.

Before.

proper relation towards the lower teeth. The articulation seems good at every point and even better than it was originally.

I have taken the liberty of bringing this case to the attention of the Institute, and shall consider myself honored if I have been able to excite interest in the minds of the members.

## Figures and Tables

**Figure f1:**
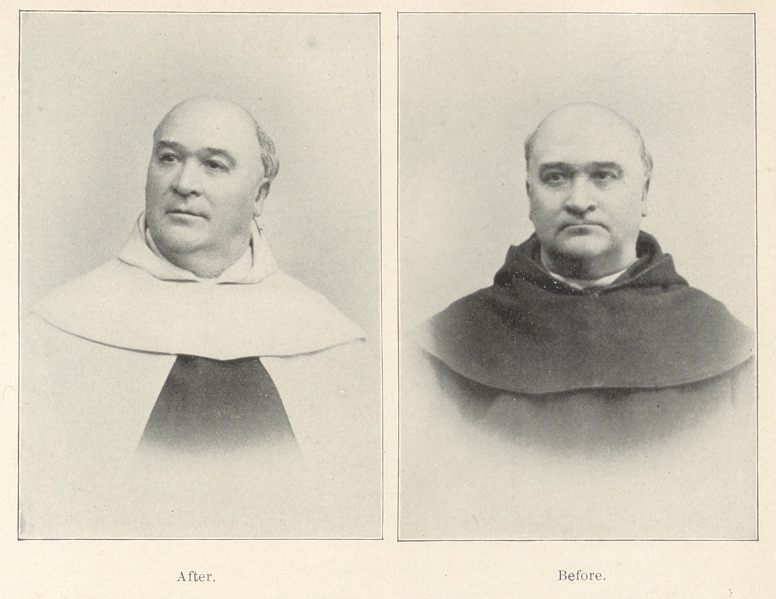


**Figure f2:**